# Supplementary material for: Reliable N-Glycan Analysis–Removal of Frequently Occurring Oligosaccharide Impurities by Enzymatic Degradation
Source: Molecules. 2023 Feb 15;28(4):1843. doi: 10.3390/molecules28041843 (PMC9967028; doi:10.3390/molecules28041843)
Supplement: Supplementary file 1 [file molecules-28-01843-s001.zip › molecules-2147360-supplementary.pdf]

Supplementary Materials

Reliable *N*-Glycan Analysis – Removal of Frequently Occurring Oligosaccharide Impurities by Enzymatic Degradation

Robert Burock [1,2,†], Samanta Cajic [1,2,†], René Hennig [1,2,*], Falk F. R. Buettner [3], Udo Reichl [1,4] and Erdmann Rapp [1,2]

[1] MPI for Dynamics of Complex Technical Systems, Sandtorstraße 1, 39106 Magdeburg; Germany
[2] glyXera GmbH; Brenneckestraße 20, 39120 Magdeburg, Germany
[3] Institute of Clinical Biochemistry, Hannover Medical School, Carl-Neuberg-Straße 1, 30625 Hannover, Germany
[4] Otto-von-Guericke University, Chair of Bioprocess Engineering, Universitätsplatz 2, 39106 Magdeburg, Germany
* Correspondence: r.hennig@glyxera.com
† These authors contributed equally to this work.

Supplementary Methods

Annotation of N-glycan Fingerprints

N-glycans were annotated by migration time matching to the glyXtool™ integrated database. Structures were confirmed by exoglycosidase sequencing using α(2-3) sialidase, α(2-3,6,8) sialidase, α(1-2,3,4,6) fucosidase and α(1-2,3,6) mannosidase (Prozyme Inc., USA), α(1-3,4) fucosidase (QA-Bio, USA), β(1-4) galactosidase and β(1-2,3,4,6) *N*-acetylglucosaminidase (New England Biolabs, Germany) as described before [1].

HILIC-UPLC-FLD Analysis of 2-AB Labeled N-glycans

2-AB labeling of N-glycans.

N-glycans were labeled for hydrophilic interaction liquid chromatography (HILIC) ultra-high performance liquid chromatography (UPLC) with fluorescence detection (FLD) (HILIC-UPLC-FLD) analysis with anthranilamide (2-AB, 2-aminobenzamide) as published by Ruhaak and coworkers [2]. Briefly, a 45 % acetic acid (AA) (v/v), a 1.05 M 2-AB, and a 3 M 2-picoline borane (2-PB) solution, all in dimethylsulfoxide, were prepared fresh before labeling. Labeling reaction was performed for 3 h @ 65 °C by mixing 50 μL of released *N*-glycan containing sample (aqueous) with 60 μL of AA/2-AB/2-PB labeling mixture (1:1:1, v/v/v). After labeling reaction the sample (110 μL) was brought to 75 % ACN_{aq} by adding 330 μL of ACN, followed by a post labeling clean-up step.

Purification of 2-AB-labeled *N*-glycans.

Excess 2-AB label, reducing agent, salts and impurities were removed from the samples using cellulose HILIC-SPE [3].Briefly, 200 μL of a 160 mg/mL microcrystalline cellulose suspension in 70:20:10 % water/ethanol/ACN (v/v/v) was filled in each needed well of a 0.45 μm GHP filter plate. Solvent was removed by application of vacuum using a vacuum manifold. All wells were washed using 5 × 200 μL water, followed by equilibration step using 3 × 200 μL ACN/water (80:20 %, v/v). Half volume of the labeled sample (220 μL of 440 μL total) was loaded onto the microcrystalline cellulose filled well and incubated 5 min @ 450 rpm. After solvent removal using the vacuum manifold, the remaining 220 μL of the sample was applied to the same well of the filter plate, incubated for 5 min, followed by a solvent removal using the vacuum manifold. All sample containing wells were subsequently washed 4 times using 200 μL 80 % ACN_{aq}. Glycans were eluted 2 twice with 100 μL of water after 5 min incubation @ 450 rpm. Combined eluates either analyzed immediately by HILIC-UPLC-FLD or stored @ –20 °C until usage.

HILIC-UPLC-FLD analysis of 2-AB-labeled N-glycans.

2-AB-labeled *N*-glycans were analyzed by hydrophilic interaction liquid chromatography (HILIC) as described by a Waters application note [4]. Briefly, fluorescently labeled N-glycans were separated by HILIC-UPLC on a Thermo Scientific™ UltiMate™ 3000 Rapid Separation Binary system (Dionex/Thermo Fisher Scientific), equipped with a Waters BEH Glycan column (particle size: 1.7 μm; column size: 150 mm length × 2.1 mm inner diameter). The instrument was under the control of

Chromeleon 7.2 (Thermo Fisher Scientific). The effluent was monitored by fluorescence detection @ an excitation wavelength of 330 nm and an emission wavelength of 420 nm. The column was held initially @ 30 % mobile phase A (50 mM ammonium formate, pH 4.4) and 70 % mobile phase B (100 % ACN). Following, a linear gradient was applied with: 0 min 70 % solvent B @ 0.561 mL/min; 1.47 min 70 % solvent B; 24.81 min 53 % solvent B; 25.5 min 30 % solvent B @ 0.4 mL/min; 29.5 min 30 % solvent B; 30.0 min 70 % solvent B @ 0.561 mL/min; 40.0 min 70 % solvent B. Samples were kept @ 10 °C in the autosampler prior to injection. 45 μL of sample in 80 % ACN (v/v) was injected. Column compartment temperature was set to 40 °C during sample measurement. The retention times of eluting glycan peaks are given in minutes.

Supplementary Tables

Supplementary Table S1: Enzyme effectivity in WS0095 buffer after 1 h. Effectivity of 10 commercially available glucoside hydrolases for degradation of maltodextrins and dextrans is compared by abundancies of mono- and oligosaccharides before and after treatment in WS0095 buffer at pH 5 for 1 h at 37 °C. Percentages of maltodextrins and dextrans were calculated after automatic peak picking with glyXtoolCE™. The most efficient enzymes for maltodextrin degradation are highlighted dark (best) and light blue (second-best). The most efficient enzymes for dextran degradation are highlighted dark (best) and light green (second-best). GAP: Glucoamylase P; DxChe: Dextranase from *Chaetomium erraticum*; DxPsp: Dextranase from *Penicillium* species. * GAP was used with a concentration of 0.17 U/µL. Incubation time was 10 min.

Substrate	Maltodextrin				Dextran			
Enzyme / Peak of	DP 1 [%]	DP 2 [%]	DP 3 [%]	≥ DP 4 [%]	DP 1 [%]	DP 2 [%]	DP 3 [%]	≥ DP 4 [%]
none	2.5	7.4	15.0	75.1	0.0	0.0	1.5	98.5
ß-Amylase	3.4	59.0	31.1	6.5	0.0	0.0	1.5	98.5
GAP*	0.0	84.8	15.2	0.0	0.0	56.9	3.1	40.0
Oligo-1,6-Glucosidase	2.8	7.3	16.7	73.2	53.5	7.1	1.3	38.1
DxChe	1.4	19.6	71.4	7.6	0.3	47.3	44.7	7.7
DxPsp	1.8	81.4	5.4	11.4	0.1	46.2	47.0	6.7
α-Amylase II-A	2.6	7.6	43.7	46.1	0.0	0.0	1.6	98.4
α-Amylase IX-A	2.4	41.4	45.1	11.1	0.0	0.0	1.4	98.6
α-Amylase XIII-A	2.3	28.2	59.0	10.5	0.0	0.0	1.3	98.7
α-Glucosidase	26.8	2.0	0.0	71.2	0.0	0.0	1.2	98.8
Oligo-α-(1,4-1,6)-Glucosidase	1.5	4.9	12.4	81.2	0.0	0.0	0.8	99.2

Supplementary Figures

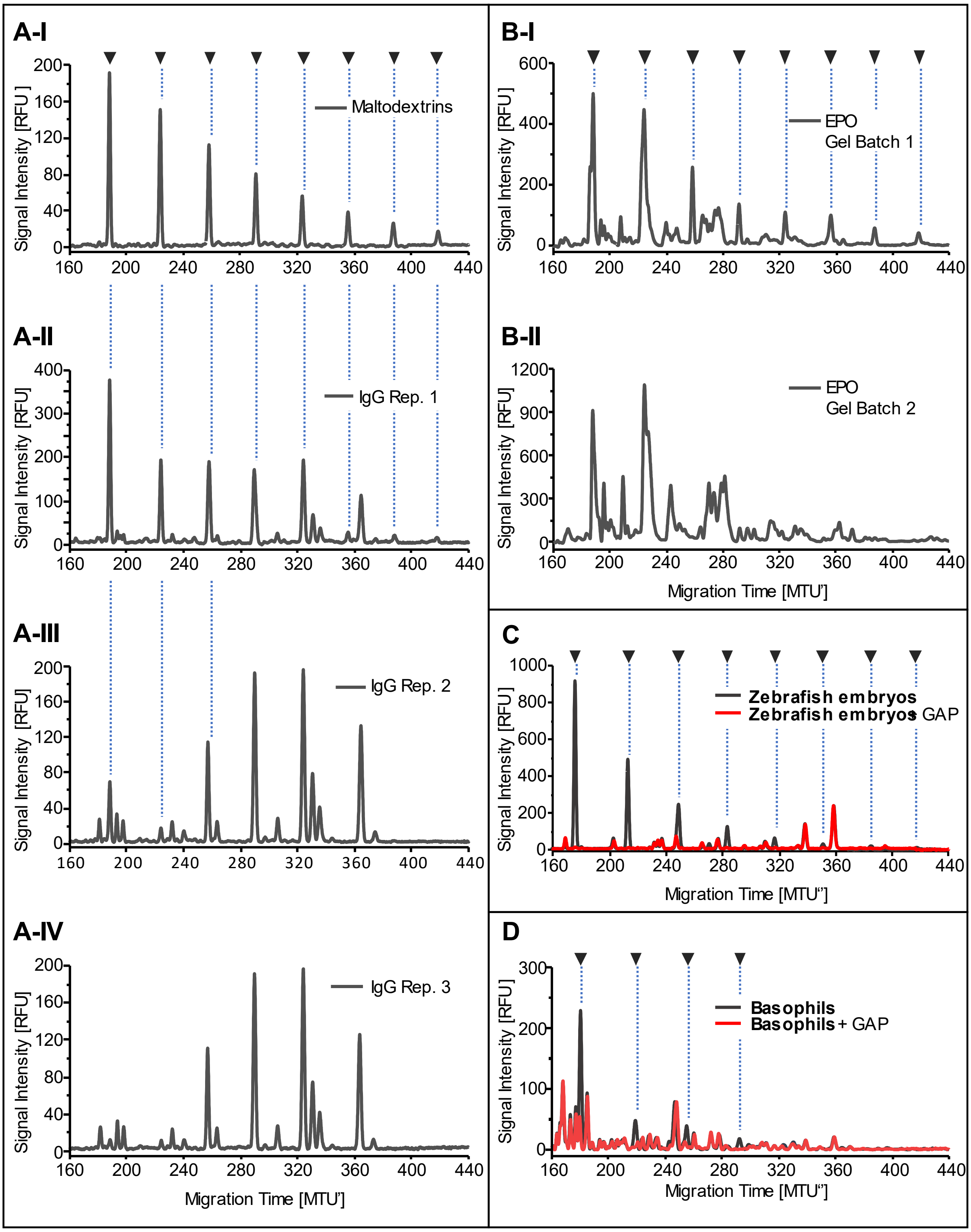

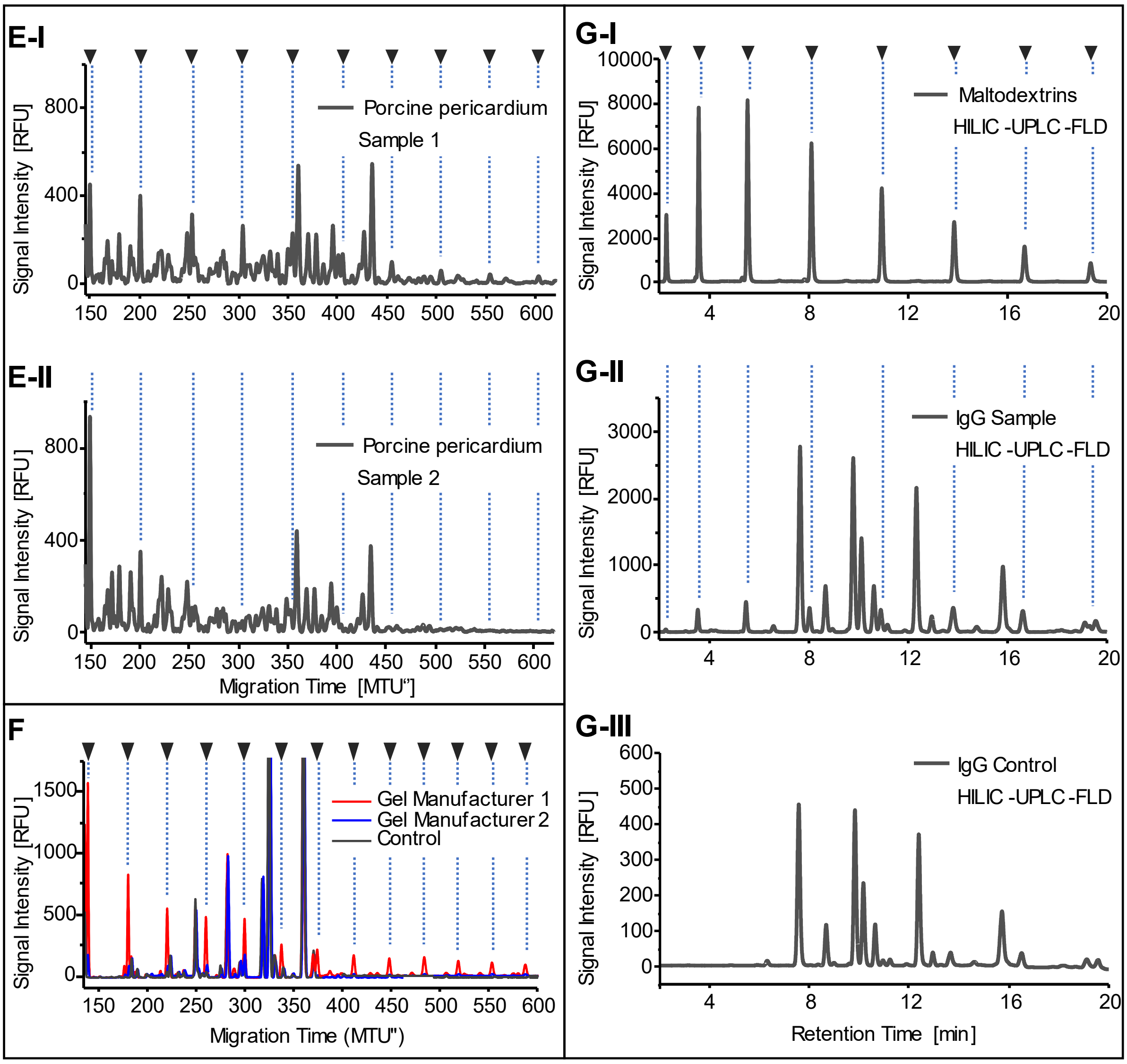

Supplementary Figure S1: xCGE-LIF and HILIC-UPLC-FLD generated *N*-glycan fingerprints of different samples with varying OSI amounts. Peaks containing OSIs are marked with ▼. Migration time plotted on the x-axis was aligned for xCGE-LIF to an internal migration time standard, resulting in Migration Time Units (MTU'). Retention times in HILIC-UPLC are given in minutes. Data analysis was performed using glyXtoolCE™.

Panel A: A-I: Fingerprint of maltodextrins (for comparison purpose). A-II to A-IV: *N*-glycan fingerprints of human IgG purified by single-step affinity chromatography from human blood plasma (replicates). While Replicate 1 (A-II) contains a high abundant OSIs, in replicate 2 (A-III) only a minor contamination was observed and in replicate 3 (A-IV), no OSIs can be found. The OSIs were identified by migration time matching as maltodextrins. All replicates were purified in one experiment from the same source material. As the contamination source the frit plate used for affinity chromatographic purification, was identified.

Panel B: *N*-glycan fingerprints of erythropoietin (EPO) after in-gel *N*-glycan release, as published in [5]. Precast SDS-PAGE gels from two different batches were used for sample preparation. While for the first batch (B-I) substantial OSIs could be detected inside the EPO-derived *N*-glycan fingerprint, the second batch (B-II) of gels, was not contaminated with OSIs and created a clean EPO-derived *N*-glycan fingerprint.

Panel C: *N*-glycan fingerprints derived from full cell lysate of zebra fish embryos. OSI contaminations inside the samples were enzymatically degraded by glucoamylase P (GAP) after recognizing a striking ladder like pattern.

Panel D: *N*-glycan fingerprint of a basophil-derived *N*-glycan sample. OSIs contaminations were enzymatically degraded by glucoamylase P (GAP).

Panel E: *N*-glycan fingerprints derived from porcine pericardium cell lysates. OSI contaminations can appear in different quantities. While for Sample 1 (E-I) contaminating OSIs show a dominant ladder pattern, OSIs inside Sample 2 (E-II) are rather inconspicuous with only one dominant peak at approx. 150 MTU'.

Panel F: Overlay of xCGE-LIF generated *N*-glycan fingerprints of IgG after in-gel *N*-glycan release, as published in [5] with an control sample not isolated via SDS-PAGE. While the control (black graph) contains no OSIs, considerable amounts of OSIs are introduced by precast SDS-PAGE gels of manufacturer 1 (red graph). Precast SDS-PAGE gels of manufacturer 2 (blue graph) introduced a low amount of OSIs.

Panel G: G-I: Chromatogram of maltodextrins (for comparison purpose). G-II: *N*-glycan chromatogram of a captured human IgG sample. G-III: A human IgG control sample (purchased standard). As the retention time of contaminating OSIs overlaps with the retention time of *N*-glycans, OSIs are also problematic in HILIC-UPLC and HILIC-UPLC based *N*-glycan analysis.

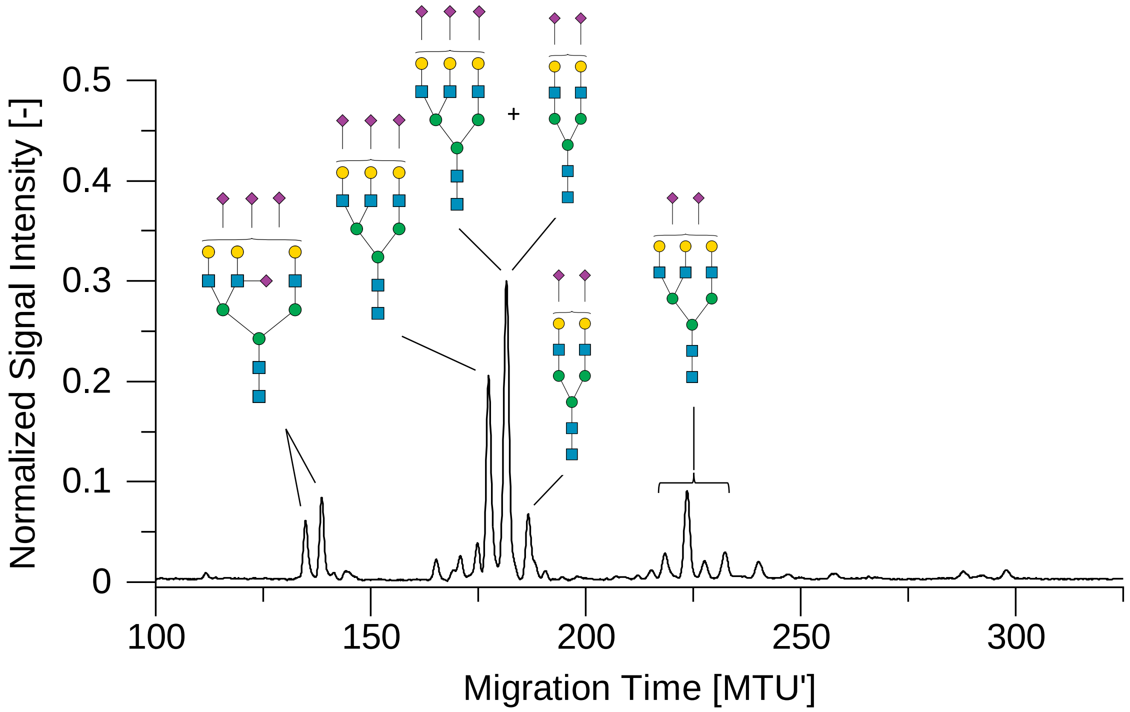

Supplementary Figure S2: Annotated xCGE-LIF generated fingerprint of bovine fetuin (bFet)-derived *N*-glycans. The annotation serves as a prerequisite for later side reaction evaluation of glucoside hydrolases. *N*-glycans of bovine fetuin were released and labeled with APTS using the glyXprepCE™ sample preparation kit (glyXera GmbH). After initial CGE-LIF analysis on a glyXboxCE™ system and *N*-glycan identification via database matching with the glyXtoolCE™ integrated database, *N*-glycan structures were confirmed by exoglycosidase treatments [1]. *N*-glycans of bovine fetuin are di- and triantennary and have a high degree of sialylation. Migration time plotted on the x-axis was aligned to an internal migration time standard, resulting in Migration Time Units (MTU'). Signal intensity (peak height) of automatically picked peaks was normalized to a sum value of 1. Data analysis was performed using glyXtoolCE™. Symbolic representation of *N*-glycan structures follows the guidelines of Symbol Nomenclature for Glycans (SNFG) [6].

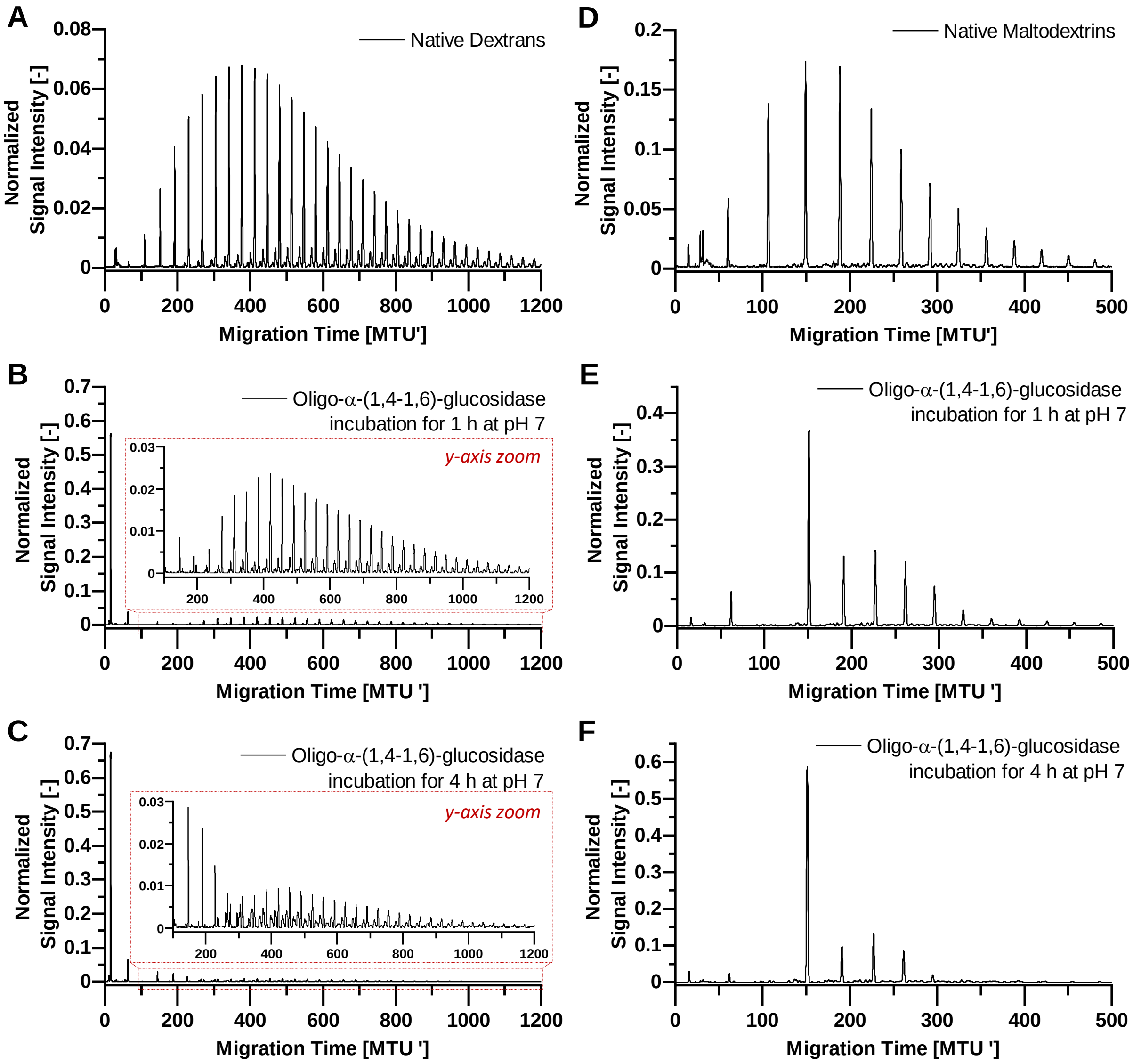

Supplementary Figure S3: Degradation of APTS-labeled dextrans (A) and maltodextrins (D) after 1 h (B and E) as well as after 4 h (C and F) with oligo-α-(1,4-1,6)-glucosidase at pH 7. Considerable amounts of these OSIs are left with ≥DP4. For digested dextrans, y-axis zoom-ins are inserted to Panel B and C. Compared to degradation with Dextranase from *Chaetomium erraticum* or glucoamylase P at pH 5, respectively, this degradation is insufficient, even though pH 7 is close to the optimal pH of 6,9 for oligo-α-(1,4-1,6)-glucosidase (stated by the supplier). Enzyme reactions proceeded in disodium phosphate-citrate buffer at pH 7 for 1 h (B and E) or 4h (C and F) as described in section 4.4. Migration time plotted on the x-axis was aligned to an internal migration time standard, resulting in Migration Time Units (MTU′). Signal intensity (peak height) of automatically picked peaks was normalized to a sum value of 1. Data analysis was performed using glyXtoolCE™.

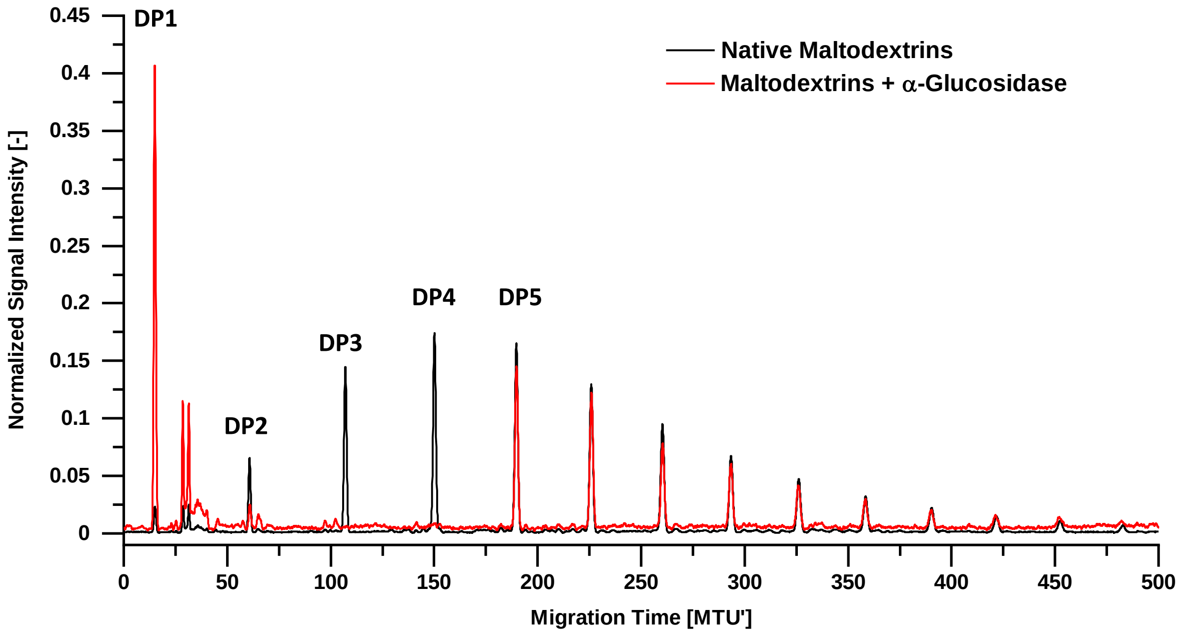

Supplementary Figure S4: Degradation of APTS-labeled maltodextrins by *α*-glucosidase. APTS-labeled maltodextrins (black graph) up to DP4 (around 150 MTU′) are selectively degraded by *α*-glucosidase which yield APTS-labeled glucose monomer (DP1, ~15 MTU′, red graph). Enzyme reaction proceeded in disodium phosphate-citrate buffer at pH 5 for 1 h as described in section 4.4. The *α*-glucosidase acted only poorly on maltodextrins with higher DP. Migration time plotted on the x-axis was aligned to an internal migration time standard, resulting in Migration Time Units (MTU′). Signal intensity (peak height) of automatically picked peaks was normalized to a sum value of 1. Data analysis was performed using glyXtoolCE™.

.

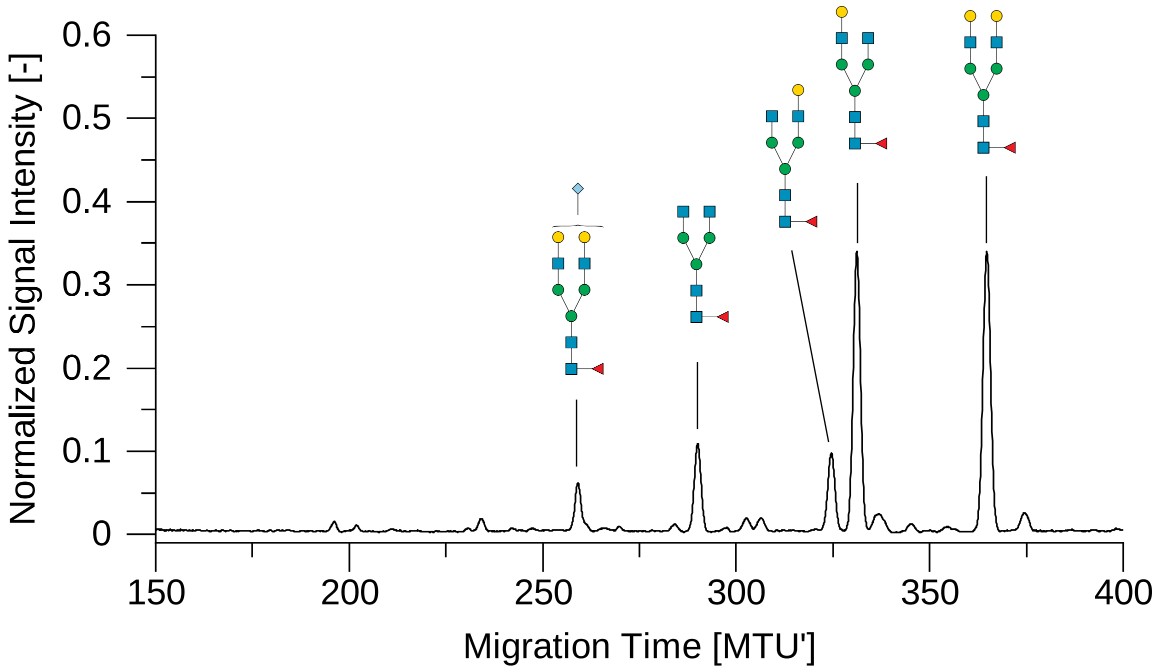

Supplementary Figure S5: Annotated xCGE-LIF generated fingerprint of bovine IgG (bIgG)-derived *N*-glycans. The annotation serves as a prerequisite for later side reaction evaluation of glucoside hydrolases. *N*-glycans of bovine IgG were released and labeled with APTS using the glyXprepCE™ sample preparation kit (glyXera GmbH). *N*-glycan annotation is based on previous bIgG *N*-glycan identification [7]. The majority of bovine IgG-derived *N*-glycans are neutral, diantennary *N*-glycans with core fucosylation. Migration time plotted on the x-axis was aligned to an internal migration time standard, resulting in Migration Time Units (MTU'). Signal intensity (peak height) of automatically picked peaks was normalized to a sum value of 1. Data analysis was performed using glyXtoolCE™. Symbolic representation of *N*-glycan structures follows the guidelines of Symbol Nomenclature for Glycans (SNFG) [2].

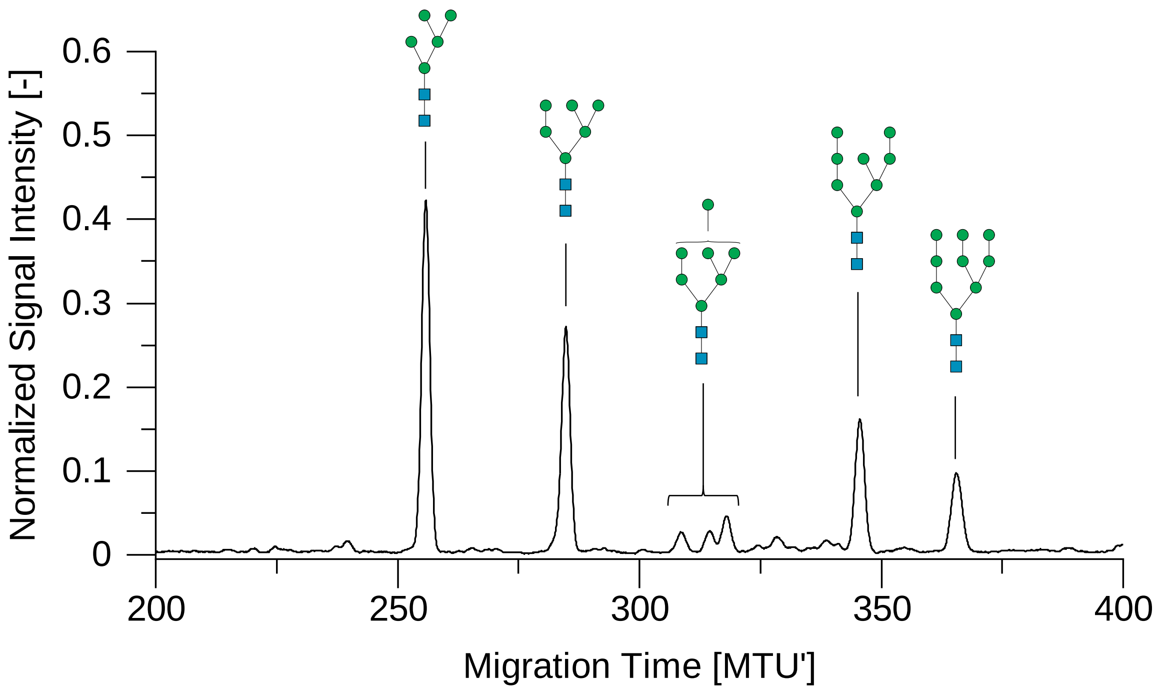

Supplementary Figure S6: Annotated xCGE-LIF generated fingerprint of bovine ribonuclease B (bRibB)-derived *N*-glycans. The annotation serves as a prerequisite for later side reaction evaluation of glucoside hydrolases. *N*-glycans of bovine ribonuclease B were released and labeled with APTS using the glyXprepCE™ sample preparation kit (glyXera GmbH). After initial xCGE-LIF analysis on a glyXboxCE™ system and *N*-glycan identification via database matching with the glyXtoolCE™ integrated database, *N*-glycan structures were confirmed by exoglycosidase treatments [1]. The majority of bovine ribonuclease B-derived *N*-glycans are of the oligomannose type. Migration time plotted on the x-axis was aligned to an internal migration time standard, resulting in Migration Time Units (MTU'). Signal intensity (peak height) of automatically picked peaks was normalized to a sum value of 1. Symbolic representation of *N*-glycan structures follows the guidelines of Symbol Nomenclature for Glycans (SNFG) [2].

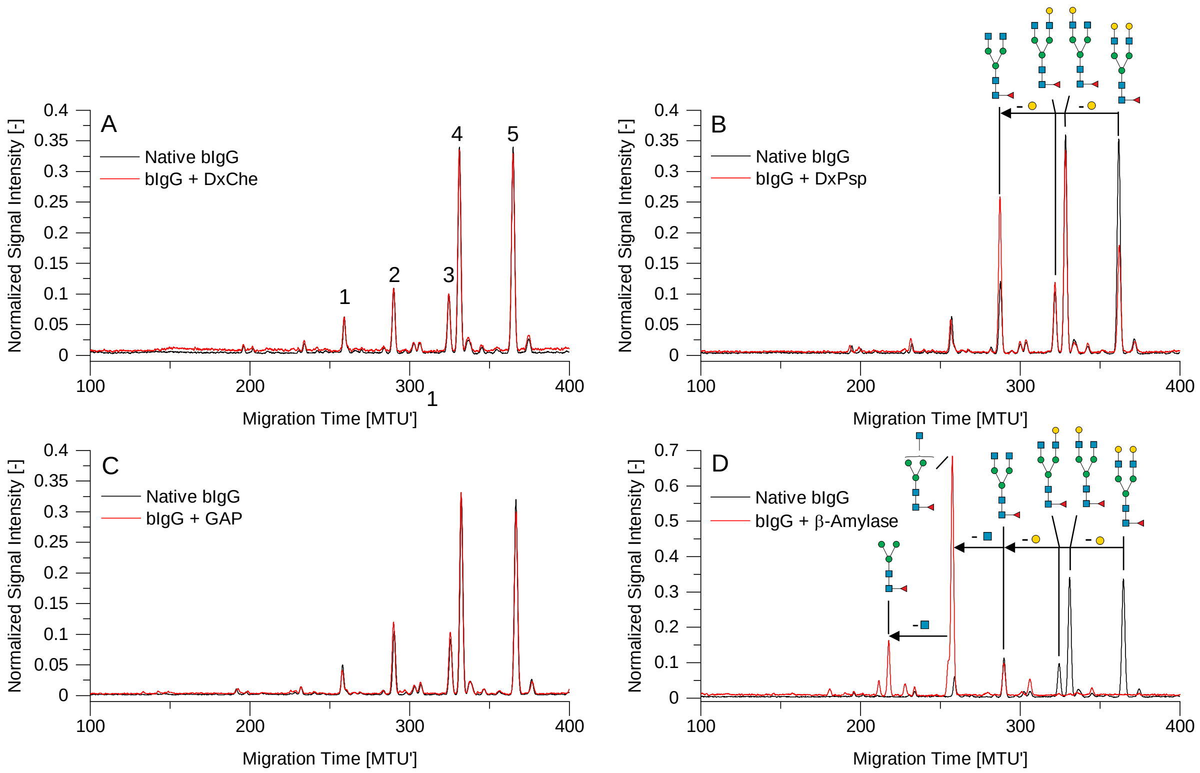

Supplementary Figure S7: Overlay of xCGE-LIF generated fingerprints of bovine IgG (bIgG)-derived *N*-glycans before and after overnight treatment with dextranase from *Chaetomium erraticum* (DxChe, A), dextranase from Pencillium sp. (DxPsp, B), glucoamylase P (GAP, 0.017 U/µL, Panel C) and β-amylase (D). β-galactosidase and *N*-acetylglucosaminidase side reactivity of DxPsp and β-amylase were revealed, but no side reactivity of DxChe and GAP could be observed. All displayed bIgG-derived *N*-glycan samples did not contain any OSIs. Migration time plotted on the x-axis was aligned to an internal migration time standard, resulting in Migration Time Units (MTU'). Signal intensity (peak height) of automatically picked peaks was normalized to a sum value of 1. The five most abundant peaks are numbered for later reproducibility assessment (Supplementary Figure 10). Data analysis was performed using glyXtoolCE™. Symbolic representation of *N*-glycan structures follows the guidelines of Symbol Nomenclature for Glycans (SNFG) [6].

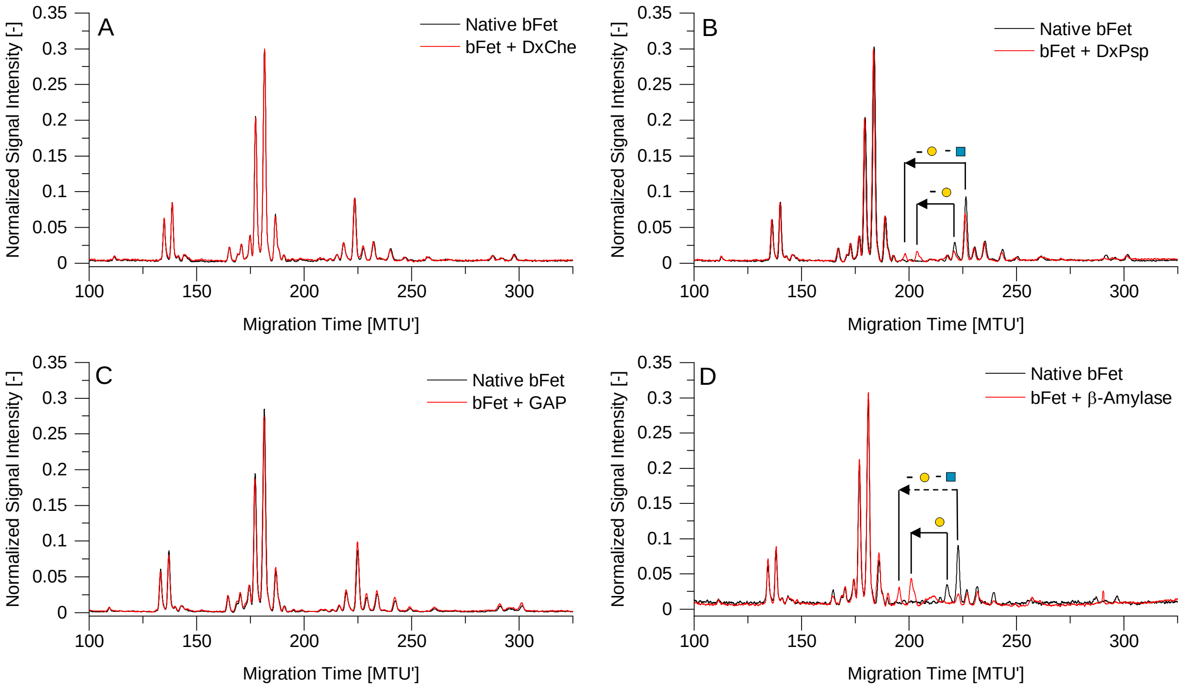

Supplementary Figure S8: Overlay of xCGE-LIF generated fingerprints of bovine fetuin (bFet)-derived *N*-glycans before and after overnight treatment with dextranase from *Chaetomium erraticum* (DxChe, A), dextranase from Pencillium sp. (DxPsp, B), glucoamylase P (GAP, 0.017 U/µL, Panel C) and β-amylase (D). β-galactosidase and *N*-acetylglucosaminidase side reactivity of DxPsp and β-amylase were revealed, but no side reactivity of DxChe and GAP could be observed. All displayed bFet-derived *N*-glycan samples did not contain any OSIs. Migration time plotted on the x-axis was aligned to an internal migration time standard, resulting in Migration Time Units (MTU'). Signal intensity (peak height) of automatically picked peaks was normalized to a sum value of 1. Data analysis was performed using glyXtoolCE™. Symbolic representation of *N*-glycan structures follows the guidelines of Symbol Nomenclature for Glycans (SNFG) [6].

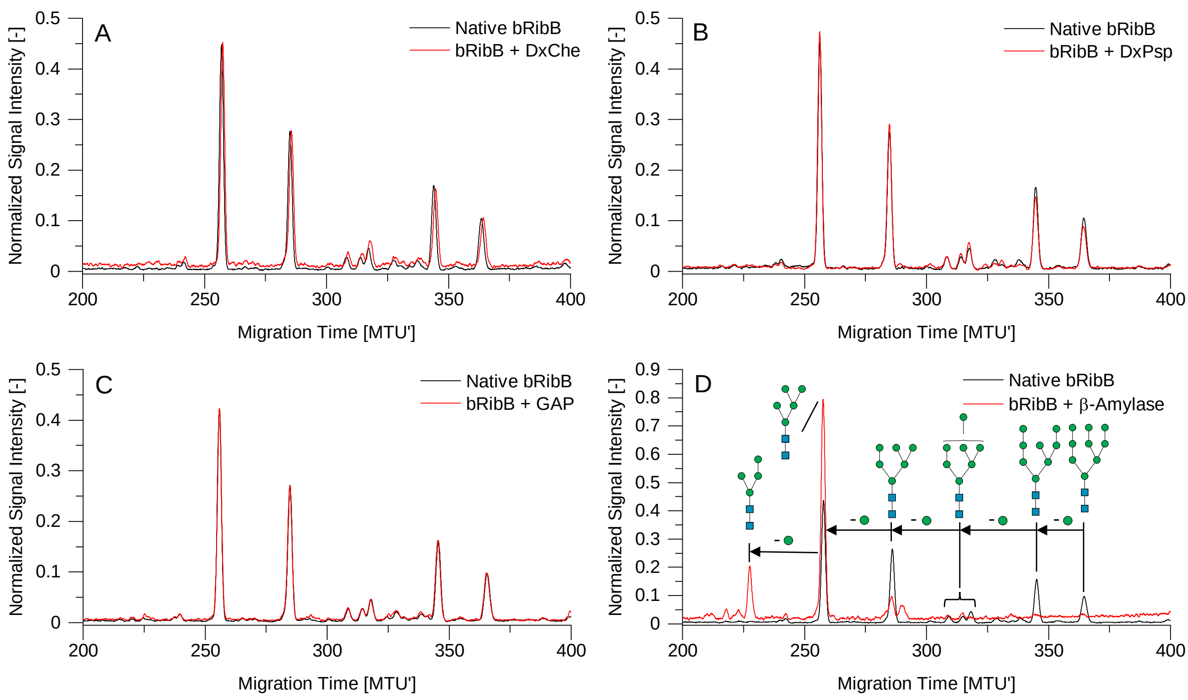

Supplementary Figure S9: Overlay of xCGE-LIF generated fingerprints of bovine ribonuclease B (bRibB)-derived *N*-glycans before and after overnight treatment with dextranase from *Chaetomium erraticum* (DxChe, A), dextranase from Pencillium sp. (DxPsp, B), glucoamylase P (GAP, 0.017 U/µL, Panel C) and β-amylase (D). *α*-mannosidase side reactivity of β-amylase was revealed, but no side reactivity of DxPsp, DxChe and GAP could be observed. All displayed bRibB-derived *N*-glycan samples did not contain any OSIs. Migration time plotted on the x-axis was aligned to an internal migration time standard, resulting in Migration Time Units (MTU'). Signal intensity (peak height) of automatically picked peaks was normalized to a sum value of 1. Data analysis was performed using glyXtoolCE™. Symbolic representation of *N*-glycan structures follows the guidelines of Symbol Nomenclature for Glycans (SNFG) [6].

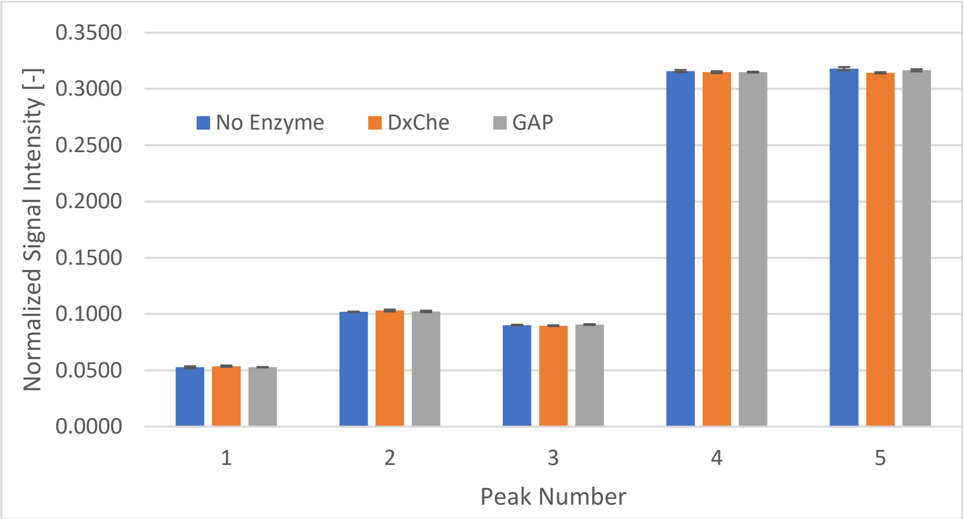

Supplementary Figure S10: Reproducibility of normalized peak heights of bovine IgG *N*-glycans with and without enzymatic treatment. Triplicates of bovine IgG were treated without enzyme (blue), with dextranase from *Chaetomium erraticum* (DxChe, orange) and glucoamylase P (GAP, 0,17 U/µL, orange) for 30 min in disodium phosphate-citrate buffer at pH 5 for 30 min. Signal intensities (peak height) of automatically picked peaks were normalized to a sum value of 1. Average values and standard deviations of the five most abundant peaks were calculated (peak numbers as included in Supplementary Figure 7A).

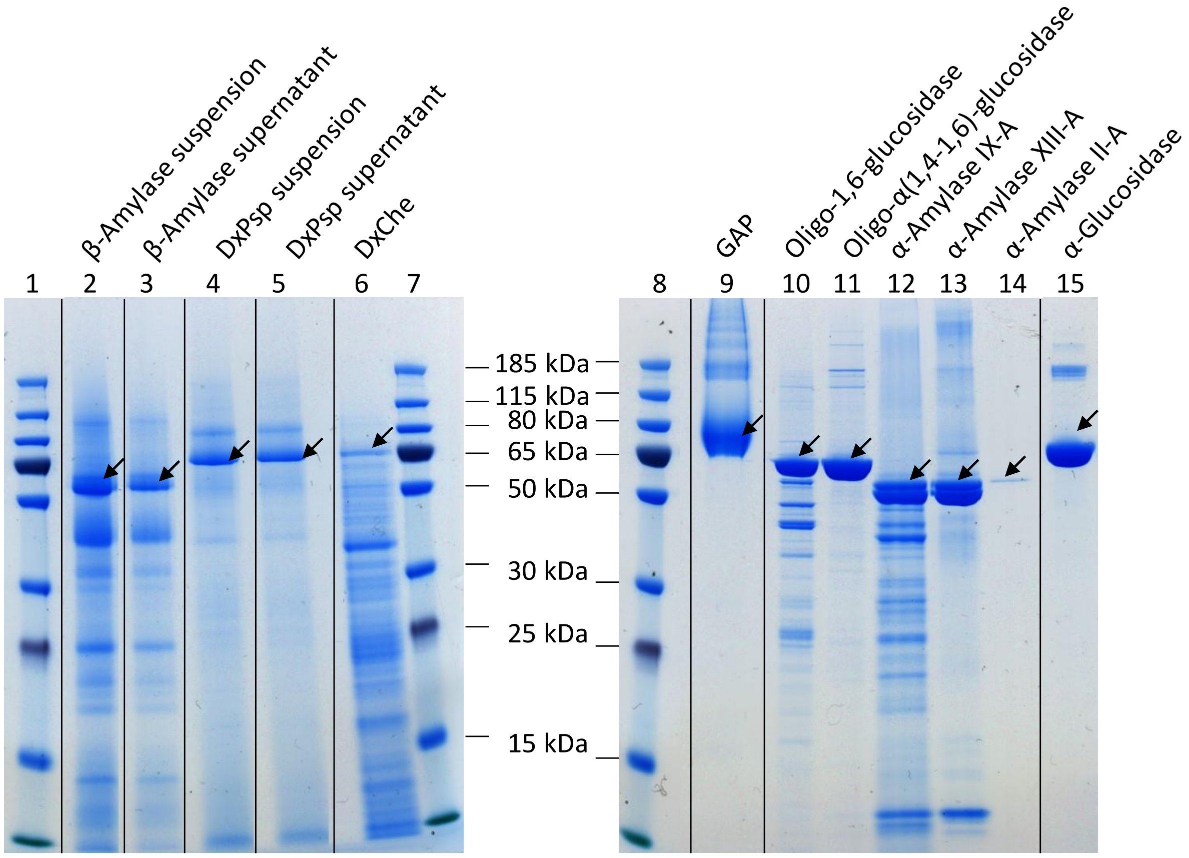

Supplementary Figure S11: Non-reducing SDS-PAGE of enzyme preparations. Complex protein band patterns are observed for β-amylase (Lane 2 and 3), dextranase from *Chaetomium erraticum* (DxChe, Lane 6), Oligo-1,6-glucosidase (Lane 10), and α-amylase IX-A (Lane 12). Additional bands not corresponding to the molecular weights of the glucoside hydrolases are seen in preparations of the dextranase from *Penicillium* sp. (Lane 4 and 5), glucoamylase P (GAP, Lane 9), Oligo-α-(1,4-1,6)-glucosidase (Lane 11). α-amylase XIII-A (Lane 13), and α-glucosidase (Lane 15). Only in the preparation of α-glucosidase (Lane 14) a single protein band can be recognized. Lanes 1, 7 and 8 contain protein size standard. If possible, solution of the commercially available enzyme preparation with 10 µg protein was applied on SDS-PAGE gels. SDS-PAGE was performed as described [5]. The enzyme formulations of the β-amylase and DxPsp were very turbid. For this reason, these enzymes were applied to the gel twice; one sample of the enzyme formulation was thoroughly homogenized (suspension), and one sample was the clear supernatant after brief centrifugation. Lane 2: β-amylase suspension. Lane 3: β-amylase clear supernatant. Lane 4: DxPsp suspension. Lane 5: DxPsp clear supernatant. Arrows indicate bands corresponding to the molecular weight of glucoside hydrolases. The gel pictures were cropped (indicated with black lines) to remove empty lanes from the depiction.

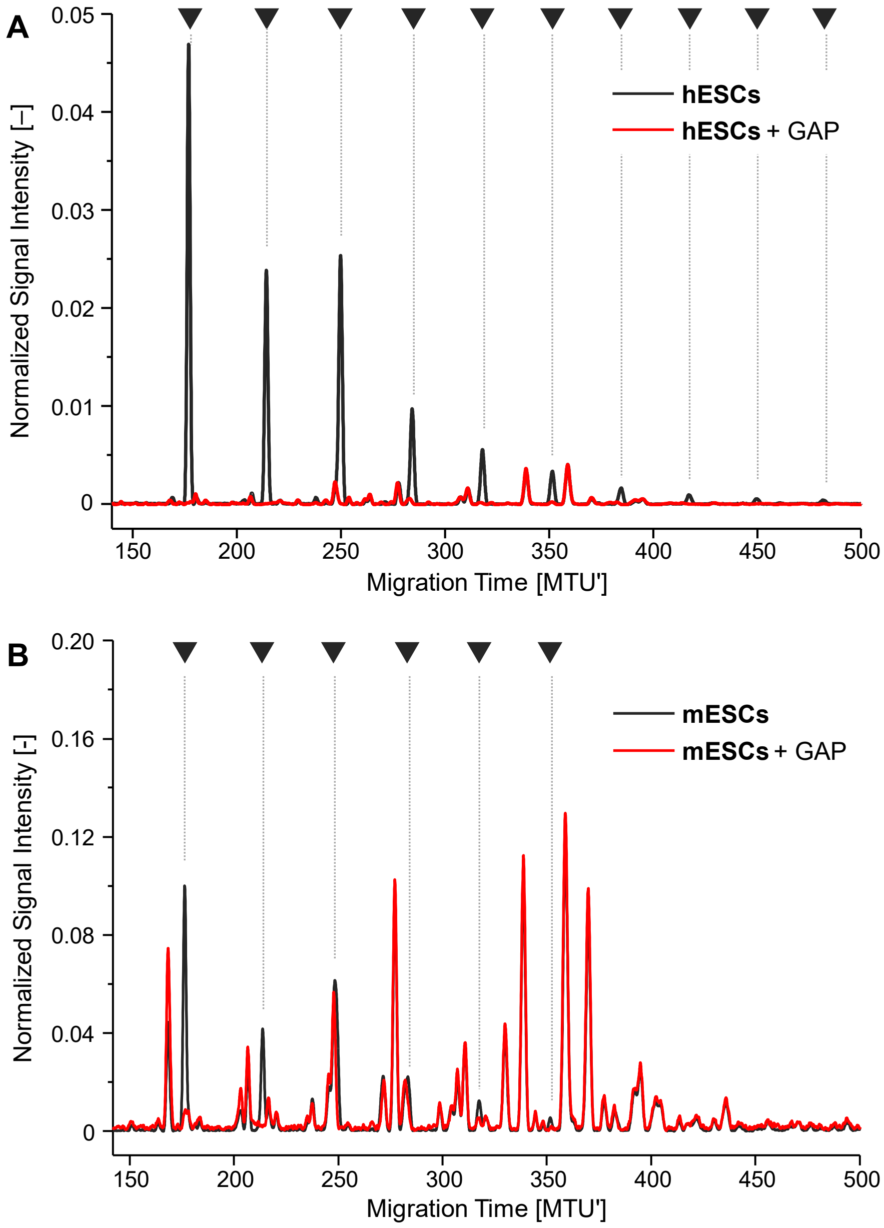

Supplementary Figure S12: Degradation of OSIs inside stem cell-derived *N*-glycan samples. The human embryonic stem cell (hESC) lysate (A, black graph) contains a high proportion of interfering OSIs with an easily recognizable ladder pattern. The murine embryonic stem cell (mESC) lysate (C, black graph) contains fewer OSIs with a pattern that cannot easily be recognized. In both examples. the enzymatic degradation of interfering OSIs obtained the unimpaired fingerprints of hESC- and mESC-derived *N*-glycans. Peaks containing OSIs are marked with ▼. Migration time plotted on the x-axis was aligned to an internal migration time standard in Migration Time Units (MTU'). Signal intensity (peak height) of automatically picked peaks was normalized to a sum value of 1. For comparison of the normalized signal intensities of *N*-glycan-derived peaks, peaks of OSI degradation products were considered for signal normalization, too. Data analysis was performed using glyXtoolCE™.

Supplementary References

1. Thiesler, C.T.; Cajic, S.; Hoffmann, D.; Thiel, C.; van Diepen, L.; Hennig, R.; Sgodda, M.; Weiβmann, R.; Reichl, U.; Steinemann, D.; et al. Glycomic Characterization of Induced Pluripotent Stem Cells Derived from a Patient Suffering from Phosphomannomutase 2 Congenital Disorder of Glycosylation (PMM2-CDG). *Mol. Cell Proteomics* **2016**, *15*, 1435–1452, doi:10.1074/mcp.M115.054122.
2. Ruhaak, L.R.; Steenvoorden, E.; Koeleman, C.A.M.; Deelder, A.M.; Wuhrer, M. 2-picoline-borane: a non-toxic reducing agent for oligosaccharide labeling by reductive amination. *Proteomics* **2010**, *10*, 2330–2336, doi:10.1002/pmic.200900804.
3. Ruhaak, L.R.; Huhn, C.; Waterreus, W.-J.; Boer, A.R. de; Neusüss, C.; Hokke, C.H.; Deelder, A.M.; Wuhrer, M. Hydrophilic interaction chromatography-based high-throughput sample preparation method for N-glycan analysis from total human plasma glycoproteins. *Anal. Chem.* **2008**, *80*, 6119–6126, doi:10.1021/ac800630x.
4. Hilliard, M.; Struwe, W.; Adamczyk, B.; Saldova, R.; Yu, Y.Q.; O'Rourke, J.; Carta, G. Development of a Glycan Database for Waters ACQUITY UPLC Systems.
5. Hennig, R.; Rapp, E.; Kottler, R.; Cajic, S.; Borowiak, M.; Reichl, U. N-Glycosylation Fingerprinting of Viral Glycoproteins by xCGE-LIF. *Methods Mol. Biol.* **2015**, *1331*, 123–143, doi:10.1007/978-1-4939-2874-3_8.
6. Varki, A.; Cummings, R.D.; Aebi, M.; Packer, N.H.; Seeberger, P.H.; Esko, J.D.; Stanley, P.; Hart, G.; Darvill, A.; Kinoshita, T.; et al. Symbol Nomenclature for Graphical Representations of Glycans. *Glycobiology* **2015**, *25*, 1323–1324, doi:10.1093/glycob/cwv091.
7. Cajic, S.; Hennig, R.; Burock, R.; Rapp, E. Capillary (Gel) Electrophoresis-Based Methods for Immunoglobulin (G) Glycosylation Analysis. *Exp. Suppl.* **2021**, *112*, 137–172, doi:10.1007/978-3-030-76912-3_4.
